# Clinical and psychological profiles of patients with different patterns of nonsuicidal self-injury

**DOI:** 10.3389/fpsyt.2025.1570880

**Published:** 2025-06-19

**Authors:** Mikhail Zinchuk, Georgii Kustov, Ilya Mishin, Sofya Popova, Ekaterina Sviatskaia, Alexander Yakovlev, Alla Guekht

**Affiliations:** ^1^ Suicide Research and Prevention Department, Moscow Research and Clinical Centre for Neuropsychiatry, Moscow, Russia; ^2^ Institute of Higher Nervous Activity and Neurophysiology, Russian Academy of Sciences, Moscow, Russia; ^3^ Neurology, Neurosurgery and Medical Genetics Department, Pirogov Russian National Research Medical University, Moscow, Russia

**Keywords:** self-injurious behavior, mental disorders, suicide prevention, psychological tests, nonsuicidal self-injury, inventory of statements about self-injury

## Abstract

**Introduction:**

Suicide remains a significant public health problem worldwide, particularly in Eastern European countries. Previous studies have shown that nonsuicidal self-injury (NSSI) is one of the most important risk factors for suicide attempts, particularly among people with mental disorders. At the same time, the risk of various dramatic outcomes, including suicide, is likely to vary among different NSSI subtypes. The aim of this study was to evaluate the relationships between NSSI parameters and clinical/psychological variables in Russian patients with non-psychotic mental disorders and suicidal ideation.

**Methods:**

The Inventory of Statements About Self-Injury-1 (ISAS) was translated and adapted in the sample of Russian patients with NSSI. The study sample consisted of 614 consecutively enrolled patients with NSSI and suicidal ideation. The data were clustered based on the method and frequency of NSSI, and the relationships between frequency and method patterns and other NSSI parameters (age at onset of NSSI, experience of physical pain during NSSI, etc.), clinical characteristics (anxiety and depression levels, psychiatric diagnosis), psychological profiles, and quality of life were evaluated.

**Results:**

Cluster analysis identified three subtypes of NSSI. Patients with a greater frequency and variety of methods of NSSI attempted suicide more often, were more clinically severe, had significantly higher scores on most pathological personality traits, had less resilience to suicide, and had a lower quality of life.

**Discussion:**

Our findings support the need for a high level of clinical attention to people with mental disorders who frequently engage in NSSI using a variety of methods. The significant differences in many of the parameters studied between the other two clusters highlighted the importance of further research into the typologization of NSSI behavior, which could lead to increased certainty in the prognosis of NSSI patients and become the basis for targeted therapy.

## Introduction

Suicide is a major public health and social problem. According to the World Health Organization (WHO), more than 700,000 people die by suicide each year, with a significant proportion of suicides occurring in young and middle-aged people (1), making suicide one of the three leading preventable causes of death among young people aged 15–29 years (2). For decades, suicide rates in post-Soviet countries have been among the highest in the European region (3, 4). Despite the large amount of research on the characteristics of suicidal behavior, most suicidologists agree that suicide prevention at the individual level is still insufficiently effective (5). One of the possible reasons for this is that there is still a gap in our knowledge of the factors that characterize those at high risk of transitioning from suicidal ideation to suicide attempt; therefore, further research in this area is warranted.

Numerous recent studies have reported the association between nonsuicidal self-injurious (NSSI) behavior and the risk of a subsequent suicide attempt (6–10). NSSI is the deliberate destruction of one’s own body tissues without suicidal intent and for socially unacceptable purposes (11). This phenomenon is widespread in both clinical samples and the general population, and several studies have shown that the number of people engaged in NSSI is increasing (12, 13). Given its high prevalence, it is particularly important to identify NSSI phenotypes that predict the development of suicidal behavior. A number of studies suggest that a longer history of NSSI, reduced pain perception and the use of multiple methods of self-harm may be associated with an increased risk of suicide (14–17). At the same time, the relationships between clinical and psychological parameters and the frequency and methods of NSSI remain understudied.

NSSI is particularly common in people with mental disorders such as borderline personality disorder (BPD) (18), eating disorders, and mood disorders (19). In addition to psychiatric disorders, NSSI has been found to be associated with certain psychological variables (e.g., personality traits such as neuroticism (20, 21)) and biological parameters (e.g., adrenocorticotropic hormone levels, altered pain thresholds) (22) as well as biographical events such as adverse life experiences (e.g., bullying, abuse) (23–25).

Previous research has attempted to identify specific patterns or subtypes of NSSI behavior through cluster analysis. The results of several studies in this area have been inconsistent, with some identifying three to four clusters based on NSSI characteristics (26), while others, identified only two distinct clusters: an “experimental NSSI” group with limited methods and frequency, and a “severe NSSI” group characterized by multiple methods and high frequency (27). Similar two-cluster solutions were reported by Bracken-Minor and McDevitt-Murphy (28), albeit with different defining characteristics. These inconsistencies in cluster solutions across studies highlight the need for further investigation of NSSI patterns, particularly in different cultural contexts.

Studies on NSSI in the Russian Federation are relatively scarce (24, 29–33). At the same time, a number of studies have shown a high prevalence of NSSI in various Russian populations (24, 30, 32). The lack of validated Russian-language instruments for assessing basic parameters of NSSI is one of the issues that needs to be addressed to stimulate further research in this area.

The Russian version of the second part of the Inventory of Statements About Self-injury (ISAS), which remains one of the most widely used instruments in NSSI research worldwide, has only recently been validated (34). The original version of this self-report questionnaire consists of two parts that are independent and can be used together (35) or as stand-alone instruments (26, 36). While the second part is designed to assess the functions of self-harm (37), the first part collects basic information about NSSI behavior, such as methods of self-harm and frequency of use of each, the respondent’s attitude toward stopping self-harming practices, etc. (27).

To date, there have been no studies on the psychometric properties of the Russian version of the ISAS-I questionnaire and on the relationships between the parameters assessed by the questionnaire and important clinical and psychological variables.

The aims of this study were: 1. to identify and describe patterns of NSSI behavior in Russian patients with non-psychotic mental disorders and suicidal ideation, and 2. to examine the relationships between the identified clusters and relevant clinical and psychological variables.

Based on previous research in this area and personal experience in treating individuals with NSSI, it was hypothesized that there would be two clusters of NSSI behaviors: one characterized by high frequency across multiple methods, and another characterized by lower frequency and fewer methods. The high frequency/multiple methods pattern was expected to be associated with a more severe clinical course, a less favorable psychological profile, and suicidality.

The first phase of the study aimed to translate and validate the ISAS-I into Russian, and then to evaluate its psychometric properties. The aim of the second phase was to assess the relationships between the frequency and method of NSSI use and clinical and psychological variables in Russian patients with non-psychotic mental disorders and suicidal ideation. Our hypothesis was that there are two subtypes of NSSI, one with a greater frequency of all methods and one with a lower frequency, and that a high frequency and greater variety of NSSI methods in Russian patients with non-psychotic mental disorders and suicidal ideation would be associated with unfavorable clinical and psychological factors and a greater risk of suicide. The study population was selected because of its clinical importance due to the simultaneous combination of three highly significant pro-suicidal factors (mental disorders, suicidal ideation and NSSI) (38).

## Participants and methods

### Procedure

The study was conducted at the Department of Suicide Research and Prevention at the Moscow Research and Clinical Center for Neuropsychiatry (MRCCN). In large cities of the Russian Federation, patients with psychotic disorders (schizophrenia, schizoaffective disorder, organic psychosis, etc.) are treated in separate clinics from those without psychotic disorders. The MRCCN specializes in the treatment of patients with non-psychotic mental disorders. The study cohort included patients with SI and NSSI aged >18 years and older identified from a consecutive cohort of patients with non-psychotic mental disorders and suicidal ideation. Patients with primary psychotic disorders, current substance use disorders, cognitive deficits below the level of comprehension on self-report scales and interviewer questions were excluded from the study.

All patients in the MRCCN on the day of admission are routinely screened for lifetime SI, suicide attempts (SA) and NSSI using the first items from the relevant sections of the Russian version of the Self-Injurious Thoughts and Behaviors Interview (SITBI) (39): “Have you ever had thoughts of killing yourself? “, “Have you ever made an actual attempt to kill yourself in which you had at least some intent to die?” and “Have you ever actually engaged in NSSI?”

On the first day of hospitalization, all patients were examined by an experienced psychiatrist who assessed the presence of exclusion criteria and diagnosed a mental disorder according to the ICD-10. All eligible patients were interviewed by the investigator to collect basic socio-demographic information and data on self-injurious thoughts and behaviors. Participants were asked to complete self-report instruments during the first two days after hospitalization. The first patient was enrolled in January 2018, and the last patient was enrolled in December 2019.

### Measures

The Inventory of Statements About Self-Injury – Part-1 (ISAS-I). The ISAS-I includes questions about the prevalence and frequency of 12 NSSI methods over the course of a lifetime: Cutting, Biting, Burning, Carving, Pinching, Pulling hair, Scratching, Banging/Hitting, Picking scabs, Rubbing skin, Sticking needles, Swallowing substances. Patients were also asked to indicate (in the “Other” section of the questionnaire) and handwrite the method they used if it was not listed above. The internal consistency of the 12 NSSI behaviors in the original study was excellent (α=0.84) (27). The questionnaire also includes items on age of onset and time since last NSSI episode, experience of physical pain during NSSI, presence of others at the time of the NSSI act, length of latency period between thinking about NSSI and acting on it, and attitudes towards stopping NSSI.

The Russian-language version of the ISAS-I was developed through forward and backward translation of the original version of the ISAS-I by two psychiatrists fluent in both Russian and English, followed by harmonization until the fullest possible correspondence between the Russian-language version and the original version was achieved. The final version of the questionnaire was prepared by a group of eight experts after a thorough analysis of several versions, taking into account the linguistic and cultural specificities of the Russian population, and is presented here in a supplementary section (**Supplementary Material S1**).

Zinchuk and colleagues (34) validated the Russian version of the Inventory of Statements about Self-Injury – Part 2 (ISAS-II), which consists of 39 items rated on a 3-point Likert scale ranging from 0 (not relevant) to 3 (very relevant). The list of items begins with an opening statement: “When I self-harm, I am…” According to the authors of the original study, the ISAS-II has two higher-order functions (Interpersonal and Intrapersonal) and 13 lower-order facets. The McDonald’s omega coefficient for the Russian version of the ISAS-II was 0.85, indicating good internal consistency. The original study found that the coefficient alphas for the interpersonal and intrapersonal scales were 0.87 and 0.80, respectively, indicating excellent internal consistency (37).

The Beck Depression Inventory (BDI) contains 21 items, each of which describes typical symptoms of depression (40). Each item is rated on a 4-point Likert scale. The Cronbach’s alpha coefficient of the Russian version of the BDI was found to be 0.86 (41).

The State-Trait Anxiety Inventory (STAI) assesses the severity of state anxiety (20 items) and trait anxiety (20 items) (42). Cronbach’s alpha was found to be −0.89 for state anxiety and −0.85 for trait anxiety (43).

The Russian version of the Personality Inventory for DSM-5 and ICD-11 Brief Form Plus-Modified (PID5BF+M), which was validated by Zinchuk et al. (44), assesses the severity of the maladaptive personality traits included in the dimensional models of personality disorders in both the DSM-5 (45, 46) and the ICD-11 (47): Negative Affectivity, Detachment, Antagonism/Dissociality, Disinhibition, Anankastia, and Psychoticism. The questionnaire contains 36 items that are scored on a 4-point Likert scale. All McDonald’s omega coefficients for the domain scores were greater than 0.80, indicating good internal consistency. The ordinal α coefficients for most facets were acceptable (α ≥ 0.70).

The Brief Reasons for Living Inventory (bRFL) was developed by Ivanoff and colleagues (48) from the original 48-item Reasons for Living Inventory (49, 50) using maximum-likelihood factor analysis. The bRFL has been found to be a reliable and valid measure of adaptive reasons for living that can be used in clinical and research settings (51). The questionnaire contains 12 statements rated on a 6-point Likert scale and assesses motives (survival and coping beliefs; responsibility to family; child-related concerns; fear of suicide; fear of social disapproval; moral objections) that prevent suicide attempts, even in the presence of suicidal ideation. The Cronbach’s alpha for the Russian version of the bRFL is −0.85 (52).

The Russian version of the World Health Organization Quality of Life Group (WHOQOL-100) instrument assesses quality of life indicators in domains such as Physical Health, Psychological Health, Level of Independence, Social Relationships, Environment, Spirituality, overall quality of life and perception of life (53). The questionnaire consists of 100 items, each of which is rated on a 5-point Likert scale. The WHOQOL-100 demonstrated high reliability in measuring quality of life in people with depression (Cronbach’s alpha = 0.96) (54). The psychometric properties of the Russian version of the questionnaire have been studied in patients with mental disorders (55).

### Data analysis

Categorical variables are presented as frequencies (%) and continuous variables as means (standard deviations). The identification of subgroups of patients with NSSI according to method/frequency parameters was performed using k-means clustering. We clustered 12 NSSI methods presented in ISAS-1. In the case of non-normally distributed data with a large number of values above the median, we decided to transform the data by ranking, i.e., assigning each patient a rank according to the frequency of manifestation of the indicator. In this transformation, the minimum value corresponds to rank 1, the next to rank 2, and so on; the maximum value corresponds to the maximum rank equal to the number of observations. When multiple values are equal, each value is assigned a rank equal to the median of the unadjusted ranks. With this transformation, the sum of the ranks for all subscales should provide an adequate assessment of the patient’s condition. The transformed data were used for cluster analysis. To validate the cluster analysis results, a functional discriminant analysis was additionally performed.

The association of clusters with categorical variables was assessed using the chi-squared test, and continuous variables were assessed using Kruskal-Wallis ANOVA with *post hoc* Dwass-Steel-Critchlow-Fligner pairwise comparisons. The Benjamini-Hochberg method was applied to control false positive results for all p-values. Statistical processing was performed in Jamovi V 2.3.17.0.

## Results

### General sample characteristics

A total of 614 consecutive patients with NSSI and SI were included in the study. Fourteen participants refused to answer the question about lifetime suicide attempts but completed all other questionnaires (for details of the enrollment procedure, see Zinchuk and colleagues (34)). The median age was 24.86 (7.86) years, and most were assigned female at birth (543 (88.4%)). Fifty-one patients (8.3%) reported an alternative gender identity.

The most common diagnoses were affective disorders (bipolar disorder – 160 (26.1%); unipolar depressive disorder – 165 (26.9%)) and personality disorders (162 (26.4%)). Fifty-five patients (9.0%) were diagnosed with more than one mental disorder. The lifetime prevalence of suicide attempts in the sample was 44.7%. The socio-demographic and clinical variables are described in more detail in our previous work on the same sample and are presented here in a supplementary section (**Supplementary Material S2**) (34).

### NSSI characteristics

None of the patients reported any difficulty in understanding the Russian version of the ISAS-I items. The most common methods of self-injury in the sample were cutting (75.2%), banging/hitting (70.8%), scratching (65.1%), picking scabs (59.4%) and biting (53.9%) (**Table 1**).

**Table 1:** Prevalence and frequency of nonsuicidal self-injury methods in the total sample.

Methods	Prevalence n (%)	Frequency			
		Mean	SD	Median	Range
Cutting	462 (75.2%)	54.01	165.59	5	0–2000
Biting	331 (53.9%)	87.07	619.32	2	0–10000
Burning	243 (39.6%)	6.91	38.19	0	0–700
Carving	228 (37.1%)	6.31	51.77	0	0–1000
Pinching	287 (46.7%)	118.96	765.48	0	0–10000
Pulling hair	254 (41.4%)	39.44	153.33	0	0–2000
Scratching	400 (65.1%)	54.72	277.58	3	0–5000
Banging/Hitting	435 (70.8%)	68.55	271.6	6	0–5000
Picking scabs	365 (59.4%)	376.44	4137.45	10	0–100000
Rubbing skin	122 (19.9%)	11.8	71.06	0	0–1000
Sticking needles	154 (25.1%)	8.35	46.49	0	0–500
Swallowing substances	154 (24.1%)	20.79	134.88	0	0–1825
Other	49 (8.0%)	31.42	451.59	0	0–11000
>1 NSSI types	578 (94.1%)				
NSSI types number		5.67	2.84	5.50	1–13

The majority of patients (94.1%) used more than one method of NSSI, and the mean number of methods used was 5.67 (2.84). Other types of NSSI reported by patients are presented here in the supplementary section (**Supplementary Material S3**).

The mean age at first NSSI was 15.3 (6.07) years. Four hundred and 72 (76.9%) patients had been engaged in NSSI in the previous 12 months. Approximately 10% of participants had never experienced physical pain while self-harming, and only 5% of patients self-harmed in the presence of someone else. For most subjects (78.8%), the time between thinking about NSSI and acting on it was less than 1 hour. Nearly one in five patients denied having a desire to stop using NSSI (**Table 2**).

**Table 2:** Characteristics of nonsuicidal self-injurious behavior in the total sample.

Variables	Mean (SD)/n (%)
Age at NSSI onset	15.3 (6.07)
12-month NSSI	472 (76.9%)
Physical pain	
Yes Sometimes No	326 (53.1%) 231 (37.6%) 57 (9.3%)
Alone when self-harm	
Yes Sometimes No	439 (71.5%) 144 (23.5%) 31 (5.0%)
Time from urge to self-harm to action	
Less than 1 hour 1–3 hours 3–6 hours 6–12 hours 12–24 hours More than 24 hours	484 (78.8%) 53 (8.6%) 9 (1.5%) 11 (1.8%) 15 (2.4%) 42 (6.8%)
Wanted to stop	
Yes No	482 (78.5%) 132 (21.5%)

### Description of the clusters

**Figure 1:**
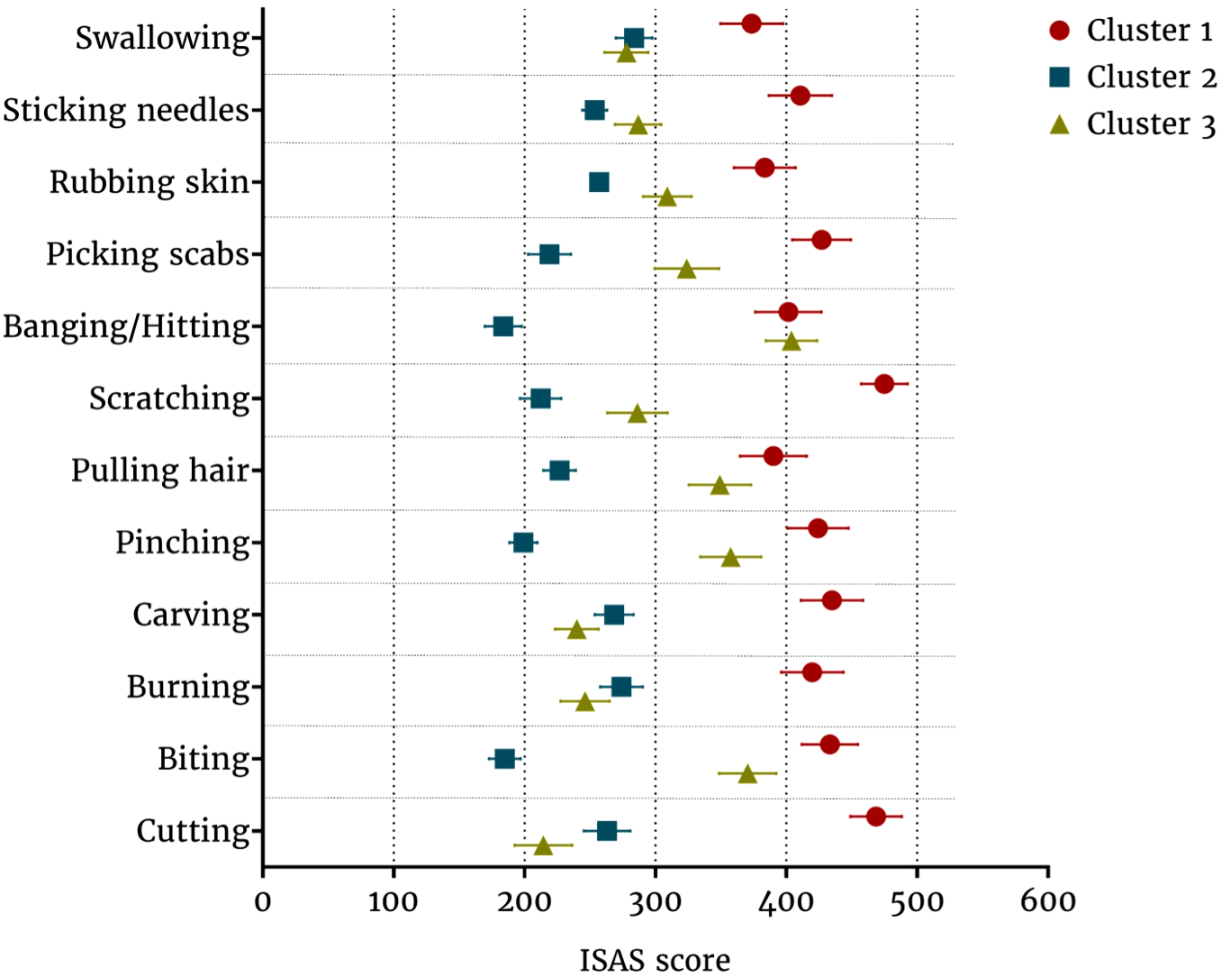
Clustering based on the type and frequency of nonsuicidal self-injury methods.

Based on the cluster analysis, three clusters were identified (**Figure 1**, **Table 3**). The first cluster included 174 patients with a high frequency of all NSSI methods and a predominance of instrumental methods of NSSI. We use the term “instrumental” to denote the self-injurious behavior inflicted by an instrument or tool, such as a blade, scissors, nails, or a burning cigarette. The term “non-instrumental” is employed to denote behaviors that involve self-inflicted harm without the use of any instrument, such as biting or hair pulling. The presentation of the latter is, in certain respects, similar to body focused behavior, yet it is distinct from it in that it is not automatic, but rather purposely inflicted for reasons delineated in the DSM-5 diagnostic criteria for NSSI disorder.

**Table 3:** Comparison of NSSI method prevalence between clusters and functional discriminant analysis results.

Methods	Prevalence n (%)				Discriminant analysis		
	Cluster 1 n=174	Cluster 2 n=267	Cluster 3 n=173	p-value	F-to-remove	Tolerance	p-value
Cutting	165 (94.8%)	202 (75.7%)	95 (54.9%)	p<0.001 χ²-72.24 df-2	39.1	0.90	p<0.001
Biting	149 (85.6%)	48 (18.0%)	134 (77.5%)	p<0.001 χ²-247.81 df-2	47.0	0.96	p<0.001
Burning	124 (71.3%)	84 (31.5%)	35 (20.2%)	p<0.001 χ²-107.49 df-2	22.2	0.95	p<0.001
Carving	126 (72.4%)	75 (28.1%)	27 (15.6%)	p<0.001 χ²-136.47 df-2	30.4	0.97	p<0.001
Pinching	135 (77.6%)	40 (15.0%)	112 (64.7%)	p<0.001 χ²-197.20 df-2	22.9	0.91	p<0.001
Pulling hair	109 (62.6%)	48 (18.0%)	97 (56.1%)	p<0.001 χ²-108.81 df-2	3.2	0.91	p=0.038
Scratching	167 (96.0%)	121 (45.3%)	112 (64.7%)	p<0.001 χ²-119.08 df-2	32.9	0.91	p<0.001
Banging/Hitting	149 (85.6%)	124 (46.4%)	162 (93.6%)	p<0.001 χ²-138.93 df-2	49.9	0.92	p<0.001
Picking scabs	149 (85.6%)	101 (37.8%)	115 (66.5%)	p<0.001 χ²-104.79 df-2	12.7	0.95	p<0.001
Rubbing skin	76 (43.7%)	10 (3.7%)	36 (20.8%)	p<0.001 χ²-105.64 df-2	1.7	0.93	p=0.179
Sticking needles	99 (56.9%)	22 (8.2%)	33 (19.1%)	p<0.001 χ²-137.35 df-2	11.3	0.93	p<0.001
Swallowing substances	79 (45.4%)	48 (18.0)	27 (15.6%)	p<0.001 χ²-53.67 df-2	4.8	0.96	p=0.007

The second cluster included 276 patients with a lower frequency of NSSI with a predominance of instrumental methods (in addition to cutting methods such as carving, burning, and sticking needles were used) and swallowing substances. The third cluster included 173 patients with a moderate frequency of self-harm and the use of non-instrumental methods (biting, pinching, hair pulling, picking scabs).

The discriminant analysis (**Table 3**) generally supported the clustering results. The overall percentage of correct classifications was high (96.1%), with similar percentages across clusters, showing a slight advantage for cluster 2 (99.3%) compared to cluster 1 (96.0%) and cluster 3 (91.3%). F-tests indicated that all variables (p < 0.05), except for rubbing skin (p = 0.179), significantly differentiated between clusters”.

### Variables associated with different clusters

A comparison of the main socio-demographic and clinical characteristics of people from different clusters is shown in **Table 4**.

**Table 4:** Comparison of the sociodemographic and clinical characteristics of patients from different clusters.

	Mean (SD)/N (%)				
Variables	Cluster 1 n=174	Cluster 2 n=267	Cluster 3 n=173	p-value	*Post-hoc* ^&^
Age*	22.26 (5.90)	26.97 (9.19)	24.20 (6.34)	p<0.001^#^ χ²-42.29 ϵ^2^-0.07	C1vsC2 <0.001 C1vsC3 = 0.001 C2vsC3 = 0.012
Sex assigned at birth**					
Male Female	10 (5.7%) 164 (93.3%)	31 (1.7%) 234 (88.3%)	30 (17.3%) 143 (82.7%)	p=0.003^#^ χ²-11.37 df-2	
Gender**					
Male Female Alternative gender identity	10 (5.7%) 143 (82.2%) 21 (12.1%)	29 (10.9%) 220 (82.4%) 18 (6.7%)	29 (16.8%) 132 (76.3%) 12 (6.9%)	p=0.007^#^ χ²-14.22 df-4	
Education level**					
Elementary and middle school High school Secondary vocational education Unfinished higher education Completed higher education	14 (8.0%) 35 (20.1%) 23 (13.2%) 73 (42.0%) 29 (16.7%)	13 (4.9%) 35 (13.1%) 42 (15.7%) 84 (31.5%) 93 (34.8%)	5 (2.9%) 32 (18.5%) 20 (11.6%) 68 (39.3%) 48 (27.7%)	p=0.001^#^ χ²-25.73 df-8	
Employment status**					
Employed Unemployed	76 (43.7%) 98 (56.3%)	125 (46.8%) 142 (53.2%)	78 (45.1%) 95 (54.9%	p=0.806 χ²-10.90 df-4	
Marital status**					
Single Married In another type of relationship (not formally married)	91 (52.3%) 10 (5.7%) 73 (42.0%)	149 (55.8%) 42 (15.7%) 76 (28.5%)	92 (53.2%) 17 (9.8%) 64 (37.0%)	p=0.003^#^ χ²-15.94 df-4	
Mental Disorder Diagnoses**					
Schizotypal disorder Bipolar disorder Major depressive disorder Anxiety disorder Obsessive-compulsive disorder Eating disorder Personality disorder Multiple psychiatric diagnoses	32 (18.4 %) 53 (30.5 %) 32 (18.4 %) 15 (8.6 %) 2 (1.1 %) 5 (2.9%) 52 (29.9%) 18 (10.3%)	24 (9.0 %) 69 (25.8 %) 78 (29.2 %) 42 (15.7 %) 5 (1.9 %) 8 (3.0%) 65 (24.3%) 24 (9.0%)	21 (12.1 %) 38 (22.0 %) 55 (31.8 %) 22 (12.7 %) 1 (0.6%) 4 (2.3%) 45 (26.0%) 13 (7.5%)	p=0.014^#^ χ²-8.52 df-2 p=0.196 χ²-3.26 df-2 p=0.010^#^ χ²-9.24 df-2 p=0.093 χ²-4.75 df-2 p=0.493 χ²-1.41 df-2 p=0.908 χ²-0.19 df-2 p=0.431 χ²-1.68 df-2 p=0.653 χ²-0.85 df-2	
Lifetime SA history**	106 (60.9%)	99 (38.4%)	65 (38.2%)	p<0.001^#^ χ²-25.02 df-2	
BDI score*	33.39 (10.24)	29.13 (10.12)	30.78 (9.97)	p<0.001^#^ χ²-15.63 ϵ^2^-0.03	C1vsC2<0.001 C1vsC3 = 0.048 C2vsC3 = 0.382
STAI-s score*	62.61 (9.51)	60.7 (10.52)	61.56 (9.76)	p=0.241	
STAI-t score*	63.16 (10.55)	60.76 (10.13)	62.99 (8.74)	p=0.007^#^ χ²-9.91 ϵ^2^-0.02	C1vsC2 = 0.010 C1vsC3 = 0.750 C2vsC3 = 0.067

*Kruskal–Wallis ANOVA; ^&^Dwass-Steel-Critchlow-Fligner pairwise comparisons; **Chi-square test; ^#^Significant after Benjamini-Hochberg correction; SA, suicide attempt; NSSI, Nonsuicidal self-injury; STAI-s, State and trait anxiety inventory – state; STAI-t, State and trait anxiety inventory – trait; BDI, Beck depression inventory.

Patients in Cluster 1 were younger than those in the other two clusters. Participants in Clusters 2 and 3 were significantly older than those in Clusters 1 and 3. The highest prevalence of males assigned at birth was found in Cluster 3, and the highest prevalence of females assigned at birth was found in Cluster 1. An alternative gender identity was significantly more common among patients in Cluster 1. Patients in Cluster 1 had a lower level of education than those in the other clusters. They were also less likely to be married than patients in the other two clusters.

Patients in Cluster 1 were significantly more likely to be diagnosed with schizotypal personality disorder and significantly less likely to be diagnosed with unipolar depression. At the same time, the severity of their depressive symptoms was significantly greater than that of patients from other clusters. Compared with patients in Cluster 2, those in cluster 1 had significantly higher levels of trait anxiety. However, no difference was found between clusters 1 and 3. The lifetime prevalence of suicide attempts was significantly higher in Cluster 1. Clusters 2 and 3 did not differ significantly on this variable.

Patients in Cluster 2 started using NSSI at a significantly older age than those in the other two clusters (**Table 3**). Compared patients in Cluster 2, significantly more patients in Clusters 1 and 3 had a 12-month history of NSSI. At the same time, there were no significant differences in these parameters between patients in clusters 1 and 3. Compared to Clusters 1 and 2, Cluster 3 was significantly less likely to report no pain when self-harming. Cluster 2 patients were significantly more likely than others to self-injure in the presence of others.

A significantly greater severity of Negative Affectivity and Detachment was found in patients from Cluster 1 compared to patients from Cluster 2. Disinhibition and Psychoticism subscale scores were significantly higher in Cluster 1 than in the other two clusters. At the same time, the level of Psychoticism was significantly lower in patients from Cluster 2 than in patients from Cluster 3 (**Table 5**).

**Table 5:** Personality profile comparison of patients from different clusters.

	Mean (SD)				
Variables	Cluster 1 n=174	Cluster 2 n=267	Cluster 3 n=173	p-value	*Post-hoc* ^&^
Negative affectivity*	3.95 (1.37)	3.51 (1.4)	3.66 (1.4)	p=0.004^#^ χ²-11.08 ϵ^2^-0.02	C1vsC2 = 0.003 C1vsC3 = 0.750 C2vsC3 = 0.067
Detachment*	2.56 (1.12)	2.13 (1.22)	2.34 (1.22)	p<0.001^#^ χ²-15.63 ϵ^2^-0.03	C1vsC2<0.001 C1vsC3 = 0.096 C2vsC3 = 0.212
Antagonism*	2.2 (1.29)	2.11 (1.27)	2.18 (1.35)	p=0.807	
Disinhibition*	3.55 (1.15)	2.67 (1.28)	3.14 (1.22)	p<0.001^#^ χ²-53.75 ϵ^2^-0.09	C1vsC2<0.001 C1vsC3 = 0.002 C2vsC3 = 0.067
Anankastia*	2.52 (1.58)	2.24 (1.49)	2.3 (1.58)	p=0.194	
Psychoticism*	3.06 (1.49)	1.9 (1.32)	2.33 (1.52)	p<0.001^#^ χ²-60.89 ϵ^2^-0.10	C1vsC2<0.001 C1vsC3<0.001 C2vsC3 = 0.014

*Kruskal–Wallis ANOVA, ^&^Dwass-Steel-Critchlow-Fligner pairwise comparisons.

Compared to those in Cluster 2, patients in Cluster 1 had a deficit of such reasons for life, as “survival and coping beliefs”, “responsibility to family”, and “child-related concerns”. At the same time, “child-related concerns” were less relevant for patients in Cluster 3 than for patients in Cluster 2. The deficit of such reasons for life as “fear of suicide” and “moral objections” was significantly more pronounced in patients in Cluster 1 than in those in the other two clusters (**Table 6**).

**Table 6:** Comparison of Reasons for Living Inventory scores among patients in different clusters.

	Mean (SD)				
Variables	Cluster 1 n=174	Cluster 2 n=267	Cluster 3 n=173	p-value	*Post-hoc* ^&^
Survival and coping beliefs*	3.67 (1.45)	4.25 (1.44)	3.96 (1.42)	p<0.001^#^ χ²-17.41 ϵ^2^-0.03	C1vsC2<0.001 C1vsC3 = 0.138 C2vsC3 = 0.081
Responsibility to family*	3.47 (1.52)	3.87 (1.71)	3.77 (1.6)	p=0.025^#^ χ²-7.39 ϵ^2^-0.01	C1vsC2 = 0.023 C1vsC3 = 0.143 C2vsC3 = 0.734
Child-related concerns*	2.61 (1.64)	3.38 (1.96)	2.82 (1.94)	p<0.001^#^ χ²-17.66 ϵ^2^-0.03	C1vsC2<0.001 C1vsC3 = 0.952 C2vsC3 = 0.005
Fear of suicide*	3.06 (1.52)	3.56 (1.69)	3.61 (1.6)	p=0.002^#^ χ²-12.37 ϵ^2^-0.02	C1vsC2 = 0.006 C1vsC3 = 0.003 C2vsC3 = 0.954
Fear of social disapproval*	2.37 (1.57)	2.46 (1.57)	2.48 (1.62)	p=0.791	
Moral objection*	1.64 (1.05)	2.32 (1.67)	2.05 (1.37)	p<0.001^#^ χ²-16.33 ϵ^2^-0.03	C1vsC2<0.001 C1vsC3 = 0.008 C2vsC3 = 0.587

*Kruskal–Wallis ANOVA, ^&^Dwass-Steel-Critchlow-Fligner pairwise comparisons.

Analysis of the WHOQOL-100 revealed significantly worse indicators of quality of life “Psychological health” in representatives of the first cluster compared to the second and third clusters. “Psychological health” score was significantly lower in patients of the third cluster compared to the second cluster. Patients in the first and third clusters had a significantly lower “Level of Independence” compared to the second cluster. “Quality of life – total” was significantly lower in the representatives of the first cluster than in those of the second cluster (**Table 7**).

**Table 7:** Comparison of quality of life parameters among patients in different clusters.

	Mean (SD)				
Variables	Cluster 1 n=174	Cluster 2 n=267	Cluster 3 n=173	p-value	*Post-hoc* ^&^
Physical health*	9.67 (2.5)	10.1 (2.51)	9.87 (2.62)	p=0.232	
Psychological*	8.06 (2.53)	9.16 (2.62)	8.51 (2.14)	p<0.001^#^ χ²-23.08 ϵ^2^-0.04	C1vsC2<0.001 C1vsC3 = 0.036 C2vsC3 = 0.038
Level of independence*	10.86 (2.79)	11.64 (2.89)	10.97 (2.64)	p=0.008^#^ χ²-9.61 ϵ^2^-0.02	C1vsC2 = 0.018 C1vsC3 = 0.909 C2vsC3 = 0.042
Social relation*	11.05 (3.24)	10.92 (3.04)	10.97 (2.84)	p=0.994	
Environment*	12.14 (2.23)	12.37 (2.35)	12.35 (2.15)	p=0.354	
Spirituality/religion/personal beliefs*	10.45 (4.14)	11.2 (4.74)	11.05 (4.06)	p=0.126	
Quality of life – total*	62.25 (11.72)	65.26 (12.05)	63.67 (10.28)	p=0.008^#^ χ²-9.57 ϵ^2^-0.02	C1vsC2 = 0.007 C1vsC3 = 0.296 C2vsC3 = 0.267
Perception of life*	9.16 (3.23)	9.7 (3.45)	9.29 (2.83)	p=0.076	

*Kruskal–Wallis ANOVA, ^&^Dwass-Steel-Critchlow-Fligner pairwise comparisons.

A comparison of the clusters according to the ISAS-II indicators showed that both intrapersonal and interpersonal motives were more pronounced in patients from Cluster 1 than in participants from Clusters 2 and 3 (**Table 8**).

**Table 8:** Comparison of NSSI-related characteristics among patients in different clusters.

	Mean (SD)/N (%)				
Variables	Cluster 1 n=174	Cluster 2 n=267	Cluster 3 n=173	p-value	*Post-hoc* ^&^
Age at NSSI onset*	13.07 (3.25)	17.02 (7.32)	14.55 (5.44)	p<0.001^#^ χ²-62.62 ϵ^2^-0.10	C1vsC2<0.001 C1vsC3 = 0.095 C2vsC3<0.001
12-month NSSI	157 (90.2%)	161 (60.3%)	154 (89.0%)	p<0.001^#^ χ²-13.41 df-2	
Physical pain**					
Yes Sometimes No	75 (43.1 %) 82 (47.1 %) 17 (9.8 %)	161 (60.3 %) 75 (28.1 %) 31 (11.6 %)	90 (52.0 %) 74 (42.8 %) 9 (5.2 %)	p<0.001^#^ χ²-22.47 df-4	
Alone when self-harm**					
Yes Sometimes No	131 (75.3 %) 40 (23.0 %) 3 (1.7 %)	189 (70.8 %) 54 (20.2 %) 24 (9.0 %)	119 (68.8 %) 50 (28.9 %) 4 (2.3 %)	p<0.001^#^ χ²-18.52 df-4	
Time from urge to self-harm to action**					
Less than 1 hour 1–3 hours 3–6 hours 6–12 hours 12–24 hours More than 24 hours	136 (78.2 %) 16 (9.2 %) 5 (2.9 %) 2 (1.1 %) 1 (0.6 %) 14 (8.0 %)	207 (77.5 %) 21 (7.9 %) 3 (1.1 %) 7 (2.6 %) 11 (4.1 %) 18 (6.7 %)	141 (81.5 %) 16 (9.2 %) 1 (0.6 %) 2 (1.2 %) 3 (1.7 %) 10 (5.8 %)	p=0.257 χ²-12.43 df-10	
Wanted to stop**	44 (25.3%)	52 (19.5%)	36 (20.8%)	p=0.337 χ²-2.176 df-2	
Interpersonal functions*	9.42 (7.57)	7.44 (6.35)	7.09 (5.52)	p=0.014^#^ χ²-8.53 ϵ^2^-0.01	C1vsC2<0.026 C1vsC3 = 0.029 C2vsC3 = 0.984
Intrapersonal functions*	18.61 (5.11)	12.39 (6.34)	14.69 (5.07)	p<0.001^#^ χ²-8.53 ϵ^2^-0.17	C1vsC2<0.001 C1vsC3<0.001 C2vsC3<0.001

*Kruskal–Wallis ANOVA, ^&^Dwass-Steel-Critchlow-Fligner pairwise comparisons, **Chi-square test.

NSSI, Nonsuicidal self-injury.

## Discussion

### Total sample characteristics

The vast majority of participants were young adults; the mean age of patients in our sample was 24.86 (7.86) years, which is consistent with previous data showing a higher prevalence of NSSI in younger age groups (56). The majority of participants were assigned female at birth (543 (88.4%)), which is also in line with data from previous studies (57, 58). However, the proportion of men in our sample was lower than in some other studies (59, 60), probably reflecting the higher use of mental health services by women in the Russian Federation (61, 62). The significant number of people with incomplete and completed higher education in our sample reflects the specificity of the Russian population and is consistent with data from the Organization for Economic Cooperation and Development (OECD), which reports that the Russian Federation ranks second out of 35 OECD member countries in the percentage of people with higher education among citizens aged 25–64 (63). Mood disorders (bipolar disorder – 160 (26.1%); major depressive disorder – 165 (26.9%)) and personality disorders – 162 (26.4%) were the most common diagnostic categories in our sample. These data are consistent with the results of a previous study conducted by Zinchuk and colleagues (24) on a Russian sample of non-psychotic patients with NSSI and SI. A more detailed discussion of the sociodemographic and clinical characteristics of the sample (data are presented in **Supplementary Material S2**) can be found in our previous work on the same sample (34).

### NSSI methods and frequency

In our sample, the most common NSSI methods were cutting (75.2%), hitting (70.8%), scratching (65.1%), picking scabs (59.4%), and biting (53.9%). The results differ somewhat from those of some previous studies. For example, in an Iranian population-based nonclinical sample, wound healing (69%), carving (34%), biting (28%), pulling hair (24%), and banging/hitting (23%) predominated (59). In a Spanish study on clinical samples, banging (63.4%), cutting (52.6%), scratching (49%), cutting superficially (39.9%), taking dangerous substances (39.7%) and biting (38.1%) were the most common methods of NSSI (60). In a population-based study of a nonclinical sample, E. Klonsky (27) reported that banging/punching was the most common form of NSSI, followed by hair pulling, pinching, cutting, and biting. The difference in the results obtained may be explained by the characteristics of our sample, which consisted of hospitalized patients with a nonpsychotic mental disorder and suicidal ideation, whereas other studies have mainly examined population samples. We assume that the high frequency of cutting, a method associated with the violation of skin integrity and cosmetic defects, reflects the severity of the patient’s condition. Our hypothesis is supported by data from studies that revealed an increased risk of suicide among those who cut themselves (64, 65). The role of so-called ‘tissue-damaging’ NSSI (those that result in bodily tissue damage) in weakening the suicide barrier through ‘habituation’ to the experience of pain and the sight of blood is confirmed by the high frequency of suicide attempts among participants in our sample (66, 67).

The high prevalence of NSSI in the past 12 months in our sample of 472 participants (76.9%) is generally consistent with previous reports (68, 69); however, because many studies used different time periods (e.g., past six months or past three months), it is not always possible to compare results across studies (70). We believe that the high incidence of current NSSI is due to the fact that all study participants had symptoms of a mental disorder severe enough to require admission to a psychiatric hospital. Similarly, a study by Andrewes and colleagues (71) of a sample of patients with BPD found a high prevalence of NSSI in the past 12 months (75.7%), with a relative increase in the frequency and severity of NSSI in the months preceding the suicide attempt. Given that eliminating negative feelings is a major reason for NSSI, the high frequency of self-harm in the past 12 months in our sample is also to be expected.

### Other NSSI characteristics from the ISAS-I

The mean age at onset of NSSI was 15.3 (6.07) years, which is consistent with previous findings of the typical mean age of onset of NSSI (14–15 years) in clinical samples (72). In addition, the relatively low percentage of participants with late-onset NSSI in our sample supports data from previous studies that earlier onset is a risk factor for recurrent episodes of NSSI and a predictor of suicide attempts (73) (among our study participants, 76.9% had self-injured in the year prior to assessment, and 45% had attempted suicide in their lifetime).

In our sample, patients were more likely to report experiencing pain while self-injuring (yes – 326 (53.1%); sometimes – 231 (37.6%)), which is not entirely consistent with literature data on increased pain tolerance among people who practicing NSSI (23). In our opinion, this could be explained by the high frequency of instrumental NSSI methods, which are associated with more severe traumatization than non-instrumental NSSI methods, which do not violate the integrity of the skin. In addition, individuals from our sample were characterized by relatively high levels of anxiety (STAI-s 61.48 (10.05)) and depression (BDI-30.08 (10.25)), which, according to a number of experimental studies, are associated with a decrease in pain sensitivity threshold (74). In general, it should be recognized that the influence of the experience of pain during an NSSI episode on the reduction of negative emotional affect and altered pain thresholds in regular self-injurers remains controversial and is largely determined by the characteristics of the study sample (75).

Only 5% of the study participants reported that they were not alone at the time of self-harm, which is lower than findings in population-based studies (approximately 21–52%) (28, 73, 76, 77). This finding is consistent with previous data on the relationship between the severity of NSSI and the preference to be alone at the time of self-harm (78). In addition, it may be explained by the predominance of intrapersonal rather than interpersonal motives for NSSI among our study participants (34).

The short duration of the mean time from the onset of thoughts about NSSI to the completion of an act of self-harm (less than 1 hour in 78.8%) may be explained by high rates of impulsivity (79), including negative urgency (the tendency to act impulsively in response to negative emotions), among people receiving inpatient psychiatric treatment (80), particularly among those with a history of suicidal or nonsuicidal self-injurious behavior (81).

Almost 80% of the study participants reported a desire to stop self-harming, which may be the result of both intrapersonal and interpersonal factors (82), such as self-stigma and perceived stigma (83, 84) associated with cosmetic defects resulting from NSSI (the most commonly used NSSI methods in our sample leave a typical scar pattern) (83, 85)).

### Sociodemographic, clinical and psychological profiles of patients from different NSSI clusters

The first cluster (Cluster 1) was characterized by a high frequency of self-harm and the use of all NSSI methods. Among patients in the second cluster (Cluster 2), the frequency of self-harm was generally lower than that in the other two clusters. Patients in this cluster used burning, carving, and sticking needles as their preferred NSSI method, which distinguishes them from patients in the other clusters. These methods were not among the most common in either of the other two clusters, and swallowing substances for nonsuicidal purposes (the most common method in Cluster 2) was among the least common in the other two clusters. Patients in the third cluster (Cluster 3) mainly used non-instrumental methods of self-injury, resulting in minor trauma to body tissues, such as rubbing skin, picking scrubs, pulling hair, pinching and biting.

Overall, the results confirm our initial hypothesis that a high frequency of NSSI and multiple self-injury methods used are associated with unfavorable clinical and psychological parameters in patients with nonpsychotic mental disorders and suicidal ideation. Patients included in Cluster 1 were characterized by greater clinical severity of mental disorders, as manifested by a higher frequency of bipolar and schizotypal disorder diagnoses, greater severity of depressive symptoms regardless of diagnosis and higher levels of trait anxiety, and a greater number of lifetime suicide attempts. Our results are consistent with previous findings in other linguistic-cultural samples (27, 86), suggesting that a greater frequency and variety of NSSI methods are associated with greater severity of mental disorders and greater suicide risk, regardless of cultural context (87–89).

Participants in Cluster 1 had significantly lower scores on the bRFL at the time of assessment, reflecting a lack of reasons to stay alive when suicidal ideation occurred (**Table 5**).

This finding is consistent with the data on suicidal behavior in our sample: more than half of the patients in Cluster 1 reported at least one suicide attempt in the past. The resilience deficit was more pronounced when Cluster 1 was compared with Cluster 2 and concerned the following domains: “Survival and coping beliefs”, “Responsibility to Family”, “Child-related Concerns”, “Fear of Suicide” and “Moral Objections”. Deficits in these areas may contribute to the emergence of suicidal thoughts in situations of psychological distress, as well as reduce the anti-suicide barrier that prevents individuals from transitioning from suicidal thoughts to suicide attempts. These beliefs are also potentially modifiable and may be targets for psychotherapeutic correction (90–92). “Moral Objections” was the least significant factor for patients in all clusters but was particularly low for participants in Cluster 1. Interestingly, the only factor that did not differ between clusters was “Fear of Social Disapproval”, which participants in all clusters rated as one of the least important deterrents to attempting suicide. In a previous study with a sample of German students and patients, Cwik and colleagues (51) found that “Moral Objections” and “Fear of Social Disapproval” were not substantially associated with parameters such as suicidal ideation, level of depression, perceived burdensomeness, social support, or positive mental health. One possible explanation for these findings is that, in secular societies such as the Federal Republic of Germany and the Russian Federation, moral objections are relevant to only a small number of young people contemplating suicide. Data on the factors of suicide resilience in Russian-speaking patients with NSSI are scarce, and to the best of our knowledge, no studies have been conducted in this area until current research. The study population most closely related to ours comes from a recent study in the Russian Federation, which examined reasons for living among psychiatric inpatients with nonbinary gender identity and suicidal ideation (93). The authors reported that “Moral Objections,” “Fear of Social Disapproval,” and “Child-related Concerns” were the three least important reasons for not attempting suicide among participants with both cisgender and non-binary gender identities. These findings are consistent with a population-based study by Tanner and colleagues (94), who found deficits in reasons for living, such as “Survival and Coping Beliefs,” in youth with NSSI who had attempted suicide, compared to those with suicidal ideation and NSSI alone. Another study by Muehlenkamp and colleagues (15) revealed that self-injurious adolescents who attempted suicide reported significantly fewer reasons for living. Our findings are consistent with previous studies indicating that the affect regulation and self-punishment functions of NSSI are strongly associated with its persistence, while the anti-suicide function is directly correlated with increased suicide risk. As shown by Szewczuk-Bogusławska and colleagues (95), these functions not only shape the trajectory of NSSI behaviors but also mediate the relationship between persistent NSSI and suicide risk, emphasizing their importance as targets for intervention.

Clusters 2 and 3 did not differ significantly from each other in terms of bRFL scores, except for the “Child-related Concerns” domain, which may be explained by the older age of participants in Cluster 2 and, consequently, their greater likelihood of having children. Interestingly, the percentage of participants who had attempted suicide at least once in their lifetime was nearly identical in Clusters 2 and 3.

According to the PID-5-BF+M, we found greater severity of negative affectivity and detachment, as well as higher scores on the Disinhibition and Psychoticism subscales, in patients in the first cluster (**Table 4**). Somma and colleagues (96) reported similar findings when assessing the PID-5 in an inpatient sample of Italian adolescents with NSSI. Their study showed that individuals with high negative affectivity scores were more than three times as likely to exhibit severe NSSI behaviors as individuals with low negative affectivity scores. Previously, Junker and colleagues (97) reported that neuroticism and psychoticism in adolescents posed a risk for future hospitalization for NSSI, while extraversion and positive self-esteem reduced this risk. Patients in Cluster 1 had the highest frequency of NSSI and higher levels of disinhibition compared to those in the other two clusters. This finding is consistent with other studies showing that people with long-term NSSI demonstrate lower levels of self-management (98, 99), persistence and cooperation as well as greater levels of novelty-seeking and harm avoidance, compared to clinical controls with mental disorders but without NSSI (99). The approach-avoidance conflict that arises from this pattern may be a cause of emotional instability, resulting in individuals being more motivated to obtain the immediate benefits of NSSI (e.g., relief from negative emotions) with less concern about the long-term consequences of NSSI. Recent clinical research supports our findings that people with the highest levels of PID-5 maladaptive traits (negative affectivity, detachment, antagonism and psychoticism) are those who have both SI and NSSI (100).

According to the “Quality of Life – General” and “Psychological Health” indicators of the WHOQOL-100, patients in Cluster 1 had a lower quality of life than patients in the other clusters, reaching a level of significance. Previous studies have shown greater dissatisfaction with mental and physical health among people who practice NSSI (101, 102). From our point of view, the lower quality of life indicators in patients in Cluster 1 are explained by the greater severity of their depression and anxiety.

Additionally, patients in Cluster 1 were characterized by several sociodemographic features, including younger age, female sex at birth, alternative gender identity, lower level of education, and lack of registered family relationships. These last two parameters may reflect the younger age of Cluster 1 patients at the time of examination or represent an unfavorable social and family outcome of a more severe mental disorder course. Previous studies have also identified a higher risk of severe NSSI among younger and unmarried individuals (103, 104).

All of the above results confirm our research hypotheses that high frequency and variety of NSSI methods are associated with adverse clinical and psychological parameters, lower quality of life, and suicide risk. However, contrary to our expectations, we obtained three clusters instead of two.

Although the frequency of NSSI was lower in Cluster 2 patients, which can be considered a protective factor, the use of instrumental methods and the use of dangerous substances, as well as an earlier age of onset of NSSI, were previously found to be important predictors of suicide risk (105–107). At the same time, patients in Clusters 2 and 3 in our study did not differ significantly in terms of previous suicide attempts. This suggests that these factors may only become pro-suicidal in the presence of specific clinical or psychological parameters, such as reasons for life deficit or a certain personality profile.

Patients in Cluster 3 exhibited moderate NSSI frequency with a predominance of non-instrumental methods. Compared to Cluster 2 patients, Cluster 3 patients were more likely to be assigned male at birth, to be younger at the time of assessment and onset of NSSI, to have injured themselves for interpersonal reasons, to have the personality trait of psychoticism, and to have a lower quality of life in domains such as the psychological domain and level of independence. In our opinion, these results support the distinction of these 3 clusters and the importance of their further investigation.

To overcome the difficulties in predicting outcomes (e.g., cessation of self-harm, suicidal behavior, etc.) in people who engage in NSSI, several studies have attempted to identify subtypes of NSSI by clustering biographical data, psychological and clinical parameters, and NSSI characteristics. In both the original study by Klonsky and Olino (27) and the study by Somer and colleagues (26), the authors identified four classes using latent cluster analysis and reported high heterogeneity of the resulting clusters. Additionally, Somer and colleagues (26) noted that the frequency and potential harm of different types of NSSI vary widely and emphasized that comparing the frequency of different types of NSSI based on direct calculation can lead to misleading results, as, for example, 10 self-cuts are not comparable to the same number of skin scratches or scab pickings.

Using a wide range of measures in their study, Case and colleagues (108) identified four classes of self-harm and found that the “mild/experimental group”, which is characterized by a low lifetime frequency of NSSI behaviors and a low number of NSSI methods, had low last year frequency rates, low scar presence, low levels of pain experienced during self-injury, and low levels of identification with ISAS functions. These individuals scored highest on protective factors and lowest on risk factors for future NSSI recurrence. This group shares many features with the group represented in Cluster 2. The “severe” group had the opposite characteristics (endorsed cutting as a primary NSSI behavior, used an average of three methods, and, relative to the sample mean, had very high lifetime and past year frequency rates, very high scar presence, very high levels of pain experienced during self-injury, and higher levels of identification with specific ISAS functions, including affect regulation, anti-suicide, self-punishment, self-care, anti-dissociation, and marking distress).

A study by Vaughn and colleagues (109) also identified four latent classes of NSSI based on childhood experiences of physical and sexual abuse, neglect, and family violence. The authors revealed that the “severe high abuse/neglect/family violence” group had high levels of clinical psychiatric and personality disorders and increased suicide risk. In contrast, the “low abuse/neglect latent” group only occasionally experimented with NSSI, but tended to exhibit no chronic psychological disorders, and exhibited a lower prevalence of substance use and criminal behavior.

A study by He and colleagues (110) identified two subgroups of NSSI in adolescents with depression. The high-risk NSSI group, was characterized by a high frequency of involvement in NSSI in the past year, involvement in a greater variety of methods used, a higher level of suicidal ideation, and a greater likelihood of having attempted suicide in the past (compared with class 2). The low-NSSI suicidal group comprised participants who were more likely to engage in non-bloody NSSI methods, such as hair pulling, intentional hitting and intentional biting of the mouth or lips and had no severe suicidal ideation. A study by Singhal and colleagues (20, 21) identified five distinct subgroups of self-injurers based on NSSI characteristics, with the multi-method and extremely severe NSSI groups showing significantly higher levels of psychological distress and emotion regulation difficulties. Members of this cluster were most likely to engage in both mild and moderate/severe methods of NSSI more than five times in the past year, with the greatest diversity of NSSI methods among the five clusters with a prevalence of hitting, cutting skin, severely scratching and pinching. In addition, these participants had an earlier onset of NSSI behavior, though no differences were observed in terms of gender affinity (111).

In a study by Shahwan and colleagues (86) NSSI was topologized into three distinct classes using latent class analysis, as assessed by the Functional Assessment of Self-Mutilation (FASM): Class 1, ‘Experimental/Mild NSSI’; Class 2, ‘Multiple functions NSSI/Low Suicide Ideation’; and Class 3, ‘Multiple functions NSSI/Possible Suicide Ideation’ (112). Individuals in Class 1 (“Experimental/Mild NSSI”) – were characterized by a low frequency of engagement in NSSI in the past year, the use of fewer NSSI methods (compared to Class 3), a low likelihood of a past suicide attempts and the shortest time of contemplation before committing NSSI. This group appears to share characteristics similar to those of our Cluster 2. Class 2 was similar to Class 1 in terms of low-frequency NSSI engagement, practicing fewer forms of NSSI, and a low likelihood of having attempted suicide. The most striking difference between Classes 1 and 2 was in the endorsement of the functions of self-harm. Participants in Class 2 showed a higher endorsement of automatic-positive (e.g., to punish oneself), automatic-negative (e.g., to stop bad feelings), and social-positive (e.g., to get attention) reasons for non-suicidal self-injury (NSSI) than participants in Class 1. The third cluster was characterized by a high frequency of NSSI engagement, participation in more than three forms of NSSI, high endorsement of all functions of NSSI, i.e., all three functions endorsed by class 2, and social-negative use of NSSI (e.g., to avoid doing something unpleasant that one does not want to do). Class 3 also differed from classes 2 and 1 in that participants were more likely to have had a longer period of contemplation before engaging in NSSI and were more likely to have had suicidal intent when engaging in self-harm.

In general, each of the above studies revealed a severe cluster of NSSI, similar to our Cluster 1, characterized by a broader repertoire of injury methods and a higher frequency of NSSI acts. Participants in this cluster have a markedly dysfunctional personality profile, a higher frequency of active NSSI, more lifetime suicide attempts, a lack of suicide resiliency factors, and, as a result, a higher suicide risk. Cluster 2 in our sample was relatively favorable, with a lower frequency of suicide attempts and more pronounced factors of resilience to suicide. Compared with patients in clusters 1 and 3, they were less likely to have engaged in NSSI in the past year, and pathological personality traits were less pronounced in this group of participants. In some respects, Cluster 2 is similar to the “safest” clusters identified in some other studies. We haven’t found any direct parallels to our Cluster 3 in previous studies. We believe it represents an intermediate position between the first and second clusters in terms of clinical severity and suicide risk. Further studies are warranted to examine whether this subtype of NSSI is specific to the Russian population or is present in other sociocultural contexts.

In summary, our study revealed two additional subtypes of NSSI besides the previously described variant characterized by a high frequency of injury, a large number of methods used, and a high risk of unfavorable outcomes. The validity of these subtypes’ delineation is strongly supported by statistical data resulting from cluster analysis, as well as by unique biographical, psychological, clinical, and NSSI-related characteristics. Our results support the idea that NSSI is a heterogeneous condition consisting of distinct subtypes that should be studied separately (e.g., instrumental vs. non-instrumental subtypes, or a subtype that uses less frequent methods, such as burning, carving, sticking needles, and swallowing substances). These study findings could inform future research aimed at investigating the predictors, protective factors, and dynamics of different subtypes.

Our study results also could be of help for practitioners providing help to NSSI patients. They highlight the importance of regular suicide risk assessment in persons with high frequency and versatility of methods of NSSI. Additionally, therapeutic interventions aimed at increasing resilience to suicide may focus on modifiable reasons for living. The Russian version of the ISAS appears to be a reliable and useful tool for collecting comprehensive information on NSSI functions and other NSSI-related parameters. This makes it the instrument of choice for Russian psychological and medical services that provide help to persons with NSSI.

### Strengths and limitations

The results of our study should be interpreted in light of its strengths and limitations.

The main strength of this study is that the relationships between NSSI characteristics (e.g., frequency and type of self-injury) and important outcomes were examined in a high-risk population (people with non-psychotic mental disorders and suicidal ideation). We used a consecutive sample from the largest Moscow clinic for non-psychotic mental disorders to avoid selection bias. Another strength of our study is that it addresses the knowledge gap about subtypes of NSSI behavior in Russian people, a rarely studied group that differs from European and Asian populations in many socio-cultural aspects. The Russian version of the ISAS-I developed in this study was found to be a reliable instrument for collecting data on NSSI behavior in Russian-speaking individuals. This will give researchers in Russia and other Russian-speaking countries access to the same tool that is widely used in NSSI studies around the world. This could be a step forward in homogenizing data on NSSI behavior from different countries. To the best of our knowledge, this is the first study to assess factors such as quality of life and suicide resilience in Russian people with NSSI.

The main limitations of this study are the inclusion of only non-psychotic patients and the Caucasian sample. Further research is therefore needed in these populations before the findings can be generalized to the whole population of patients with mental disorders, NSSI and suicidal ideation.

## Conclusion

The Russian version of the ISAS-I is a valid tool for assessing features of NSSI in people with non-psychotic mental disorders at risk for suicide. Patients with a pattern of high frequency and versatility of NSSI methods are at higher risk of suicide compared to those with other patterns. Higher numbers of NSSI episodes and methods used are associated with a less favorable clinical profile, deficits in suicide resilience, and lower quality of life. Two other patterns of NSSI identified in our study differed in terms of sociodemographic parameters, clinical characteristics, and psychological profiles. Our findings highlight the importance of further research into the typologization of NSSI behavior, which could lead to increased certainty in the prognosis of NSSI patients and become the basis for targeted therapy.

## Data Availability

The raw data supporting the conclusions of this article will be made available by the authors upon request.
