# Supplementary material for: Clinical and psychological profiles of patients with different patterns of nonsuicidal self-injury
Source: Front Psychiatry. 2025 Jun 19;16:1570880. doi: 10.3389/fpsyt.2025.1570880 (PMC12225643; doi:10.3389/fpsyt.2025.1570880)
Supplement: Supplementary file 1 [file SupplementaryFile1.docx]

**Supplementary 1: Russian version of the Inventory of Statements About Self-Injury**

**Опросник утверждений о самоповреждающем поведении**

**Citing:** Zinchuk M, Kustov G, Popova S, Mishin I, Voinova N, Gersamija A, Yakovlev A and Guekht A (2023) Functions of nonsuicidal self-injurious behavior in Russian patients with suicidal ideation. Front. Public Health. 11:1270944. doi: 10.3389/fpubh.2023.1270944

**Supplementary 2: Sociodemographic and clinical characteristics of the sample.**

|  | N (%)/mean (SD) |
| --- | --- |
| Age | 24.86 (7.86) |
| Sex  Male  Female | 71 (11.6%)  543 (88.4%) |
| Gender  Male  Female  Alternative gender identity | 68 (11.1%)  495 (80.6%)  51 (8.3%) |
| Education level  Elementary and middle school  High school  Secondary vocational education  Unfinished higher education  Completed higher education | 32 (5.2%)  102 (16.6%)  85 (13.8%)  225 (36.6%)  170 (27.7%) |
| Employment status  Employed  Unemployed | 379 (45.4%)  335 (54.6%) |
| Marital status  Single  Married  In another type of relationship (not formally married) | 343 (52.9%)  69 (11.2%)  213 (34.7%) |
| Mental Disorder Diagnoses  Schizophrenia spectrum disorder  Bipolar disorder  Major depressive disorder  Anxiety disorder  Obsessive-compulsive disorder  Eating disorder  Personality disorder  Multiple psychiatric diagnoses | 77 (12.5%)  160 (26.1%)  165 (26.9%)  79 (12.9%)  8 (1.3%)  17 (2.8%)  162 (26.4%)  55 (9.0%) |
| Lifetime history of Suicide attempts | 270 (44.7%) |
| Beck Depression Inventory score | 30.81 (10.25) |
| State Anxiety score | 61.48 (10.05) |
| Trait Anxiety score | 62.07 (9.93) |

**Supplementary 3: Other types of NSSI reported by patients.**

|  | **Types of NSSI reported as “other”** | **Number of episodes (lifetime)** |
| --- | --- | --- |
|  | Breaking nails | 500 |
|  | Sleep and food deprivation | 50 |
|  | Prick the fingers with a needle until the blood flows, then squeeze the blood out | 30 |
|  | I tore out eyelashes and eyebrows to the point of pain, scratched skin to the point of bleeding | 300 |
|  | Pinched limbs to prevent blood flow | Number not specified |
|  | Squeezing pimples and pulling crusts off my lips, pulling hair out of my legs when I'm nervous. | 500 |
|  | Choking, pulling on a limb to the point of numbness or bruising | 300 |
|  | Choke myself | 7 |
|  | Drank boiling water | 10 |
|  | Squeezed pimples, scratched the face | 600 |
|  | Dig nails into the palms of my hands | 200 |
|  | Scratches the skin (tears off pieces of skin) | 11000 |
|  | Burning my hands with boiling water | 10 |
|  | I held hot cutlery | 10 |
|  | I twisted my fingers backwards | 15 |
|  | Beat myself | 60 |
|  | Strangling with hands or belt | 15 |
|  | I press my nails into the palm of my hand | 20 |
|  | I bite my lip and cheek, peeling the skin from my lips | 500 |
|  | Skin picking and scratching around nails | 500 |
|  | Scratched the skin to the blood | 30 |
|  | Digging nails into the palms of my hands when I'm angry | 50 |
|  | I intentionally burn my skin chemically | 3 |
|  | Broke my heel kicking the floor during an argument with my partner | 1 |
|  | Mild self-biting | 500 |
|  | Slapping and spanking myself | 5 |
|  | Self-inflicted frostbite | 100 |
|  | Enduring starvation for self-punishment | 100 |
|  | Eardrum damage | 1 |
|  | Cut off the skin | 50 |
|  | Plucked hair from the body | 500 |
|  | Starving | 2 |
|  | I bite my lips | Permanently |
|  | Wash in very hot, almost boiling water | Permanently |
|  | I slept naked on the bare floor, forbidding myself a blanket, pillow and sheets | 2 |
|  | Tattooed myself | 12 |
|  | Choke myself | 100 |
|  | I vomited | 100 |
|  | Smoked a lot until filling bad, while being a non-smoker | 1 |
|  | Self-piercing | 10 |
|  | Tattooed myself | 4 |
|  | Slightly scratched my hands | 2 |
|  | I hit the heart area with my fist | 10 |
|  | Picking pimples that no one can see | 1000000 |
|  | Picking pimples on areas of the body that are invisible to other people | 100 |
|  | I bumped into the wall | 500 |
|  | Trimmed my toenails until they bled, cutting deep into the skin around the nails | 500 |
|  | Cut off pieces of skin with scissors | 1 |
|  | Made my home animals angry, to made them bite and clawed me | 100 |
